# Neoadjuvant chemotherapy with albumin-bound paclitaxel plus cisplatin and capecitabine for locally advanced esophageal squamous cell carcinoma: a phase 2 clinical trial

**DOI:** 10.1097/JS9.0000000000002375

**Published:** 2025-05-12

**Authors:** Wen Zhang, Xiaowei Chen, Liyan Xue, Zhichao Jiang, Dong Qu, Zhaoyang Yang, Jianjun Qin, Zhen Wang, Miaomiao Zhang, Yong Li, Aiping Zhou, Shugeng Gao

**Affiliations:** a Department of Oncology, National Cancer Center/National Clinical Research Center for Cancer/Cancer Hospital, Chinese Academy of Medical Sciences and Peking Union Medical College, Beijing, China; b Department of Thoracic Surgery, National Cancer Center/National Clinical Research Center for Cancer/Cancer Hospital, Chinese Academy of Medical Sciences and Peking Union Medical College, Beijing, China; c Department of Pathology, National Cancer Center/National Clinical Research Center for Cancer/Cancer Hospital, Chinese Academy of Medical Sciences and Peking Union Medical College, Beijing, China; d Department of Imaging Diagnosis, National Cancer Center/National Clinical Research Center for Cancer/Cancer Hospital, Chinese Academy of Medical Sciences and Peking Union Medical College, Beijing, China; e Department of Comprehensive Oncology, National Cancer Center/National Clinical Research Center for Cancer/Cancer Hospital, Chinese Academy of Medical Sciences and Peking Union Medical College, Beijing, China

**Keywords:** albumin-bound paclitaxel, esophageal squamous cell carcinoma, neoadjuvant chemotherapy, pathological complete response

## Abstract

**Background:**

Neoadjuvant chemotherapy is recommended for locally advanced esophageal squamous cell carcinoma (ESCC), but more effective regimens are needed to improve the outcomes. This study evaluates the efficacy and safety of neoadjuvant chemotherapy with albumin-bound paclitaxel plus cisplatin and capecitabine (APC regimen) for locally advanced ESCC.

**Materials and methods:**

This prospective single-center phase 2 clinical study involved patients with locally advanced ESCC (T2-4aN±M0) from May 19, 2020 to September 1, 2022. Patients received neoadjuvant chemotherapy with APC regimen for four cycles and then underwent radical esophagectomy. The primary endpoints were the pathologically complete response (pCR) rate. The secondary endpoints were the major pathological response (MPR) rate, radical resection rate (R0 resection rate), disease-free survival (DFS), event-free survival (EFS), overall survival (OS), and safety.

**Results:**

Eighty-two patients with locally advanced ESCC were enrolled in the trial. Of the 80 patients who underwent surgery, the R0 resection rate was 100%, and 23 patients achieved pCR, with a pCR rate of 28.7% (95% confidence interval [CI]: 18.8%–38.6%). Fifty patients achieved MPR, with an MPR rate of 62.5% (95% CI: 51.9%–73.1%). The most common grade 3–4 treatment-related adverse events were neutropenia (25.6%), leukocytosis (14.5%), nausea (7.5%), and peripheral neurotoxicity (5.5%). Five patients developed complications within 1 month after surgery, including pneumonia (five patients, 6.3%), anastomotic fistula (one patient, 1.3%), and laryngeal recurrent nerve paralysis (one patient, 1.3%), all of which were grade 2. The local recurrence was observed in 8 (8.5%) patients, and distant metastasis in 10 (12.2%) patients. The 3-year DFS rate was 81.2%, the 3-year EFS rate 77.8%, and the 3-year survival rate 90%.

**Conclusion:**

Neoadjuvant treatment with APC regimen for locally advanced ESCC achieved excellent pCR rates and a well-tolerated safety profile. This combination chemotherapy could serve as one optional neoadjuvant treatment. (ClinicalTrials.gov: NCT04390958).

## Introduction

Esophageal cancer (EC) is the 11th most common cancer and the 7th leading cause of cancer deaths worldwide, with an estimated 511 000 new cases and 445 000 deaths globally in 2022^[^1^]^. With an annual new diagnosis of more than 224 000, EC is characterized as the seventh most common cancer in China, where it is a highly lethal disease with more than 187 500 deaths per year, among which esophageal squamous cell carcinoma (ESCC) accounted for 90%^[^2^]^. In recent years, with the advantage of comprehensive treatment strategies, the prognosis of both locally advanced and metastatic EC has improved. Approximately two-thirds of patients with EC are diagnosed at a locally advanced stage, for which neoadjuvant chemoradiotherapy remains the standard care and chemotherapy alone is also recommended for ESCC. The optimal neoadjuvant treatment paradigm remains to be established.

The pathologically complete response (pCR) rate of concurrent chemoradiotherapy can reach 43.2%–49% and the 5-year overall survival (OS) rate after surgery is about 60%, which is always recommended as the preferred choice for locally advanced ESCC^[^3–5^]^. As the main cause of failure, the incidence of distant metastasis was 25.3%–40% in the treatment modality of concurrent chemoradiotherapy followed by surgery^[^5–7^]^, it is likely that the intensity and duration of perioperative chemotherapy may be insufficient. Furthermore, in some studies, concurrent chemoradiotherapy not only achieved good results, but also increased adverse reactions. As reported by Yang *et al*, the incidences of postoperative complications such as arrhythmia (13% vs. 4.0%; *P* = 0.001) as well as pretreatment mortality (2.2% vs. 0.4%; *P* = 0.212) were higher in the nCRT group over surgery alone^[^8^]^.

In recent years, several studies have shown that neoadjuvant chemotherapy alone achieved comparable OS times with concurrent chemoradiotherapy, although the pCR rate was relatively lower. In the early JCOG 9907 phase 3 trial, neoadjuvant chemotherapy using a combination of cisplatin and 5-fluorouracil (CF) achieved a 5-year OS rate of 55%; however, the reported pCR rate was less than 5%, and limited efficacy was observed in cStage III and cT3 disease^[^9^]^. More effective regimens are therefore urgently desired and some taxane-containing doublet or triplet regimens have been developed. The pCR rate of a paclitaxel-based doublet regimen (paclitaxel 135 mg/m^2^ combined with cisplatin 75 mg/m^2^, administered on day 1 every 21 days) was reported to be only 4.7% in the ESCORT-NEO study^[^10^]^. Similarly, in phase 2 studies, docetaxel combined with CF (DCF) or a modified DCF regimen achieved pCR rates of 17% and 18%, respectively^[^11,12^]^. Another study reported a pCR rate of 24.1% for a two-cycle regimen of paclitaxel combined with CF (paclitaxel 100 mg/m^2^ and cisplatin 60 mg/m^2^ on day 1, with a continuous intravenous infusion of 5-fluorouracil 700 mg/m^2^ per day for 5 days). However, the 5-year OS rate for this regimen was only 22%, and the study did not compare outcomes with concurrent chemoradiotherapy^[^13^]^. The recently published JCOG1109 phase 3 trial directly compared neoadjuvant concurrent chemoradiotherapy with various neoadjuvant chemotherapy regimens^[^6^]^. In this study, the pCR rate of concurrent chemoradiotherapy with 5-fluorouracil and cisplatin in ESCC was much higher than neoadjuvant chemotherapy alone with DCF or with CF (38.5%, 19.8%, and 2%, respectively), while no superior 3-year OS rate was observed (68.3%, 72.1%, and 62.6%, respectively). However, the DCF regimen was associated with incidence of high-grade toxicity. Even the modified DCF regimen resulted in grade 3/4 neutropenia and febrile neutropenia rates of 56% and 20%, respectively^[^12^]^. Although neoadjuvant chemotherapy has been recommended for preoperative treatment of locally advanced ESCC, there is still a need to explore more efficient and better-tolerated chemotherapy regimens to further increase the pCR rate and improve long-term survival.

The introduction of immune checkpoint inhibitors (ICIs) has improved the pCR rates of neoadjuvant systemic treatment for the treatment of locally advanced ESCC. In several studies, the pCR rates of neoadjuvant treatment combined with an ICI were 8%–56%^[^14^]^. As a result, this strategy is expected to be a promising preoperative neoadjuvant treatment modality for locally advanced ESCC, but the benefits of long-term survival and safety still warrant verification. Currently, a randomized phase 3 study comparing chemotherapy combined with ICIs with concurrent chemoradiotherapy is underway, which is expected to finally clarify its role^[^15^]^. In current studies, chemotherapy regimens combined with immunotherapy predominantly include taxane- and platinum-based agents. It has been suggested that the use of corticosteroids may potentially impact the efficacy of ICIs. Since both paclitaxel and docetaxel often require corticosteroid premedication, albumin-bound paclitaxel may offer a distinct advantage as a partner for ICIs due to its lack of requirement for steroid premedication, thereby potentially preserving the effectiveness of immunotherapy^[^16^]^.

**HIGHLIGHTS**

- In this prospective single-center phase 2 study that included 82 adults receiving neoadjuvant chemotherapy with APC regimen for four cycles and radical esophagectomy, the pCR rate was 28.7% (95% confidence interval [CI]: 18.8%–38.6%).
- The most common grade 3–4 treatment-related adverse events were neutropenia (25.6%), leukocytosis (14.5%), nausea (7.5%), and peripheral neurotoxicity (5.5%).
- Neoadjuvant treatment with APC regimen is highly active with excellent pCR rates and promising survival with a well-tolerated safety profile in patients with locally advanced ESCC.

Albumin-bound paclitaxel is characterized by good solubility, high concentration in tumor tissue, as well as the advantage of not requiring corticosteroid premedication, and has been widely adopted for the treatment of advanced gastric and pancreatic cancer. Capecitabine, an oral derivative of 5-fluorouracil, also offers the convenience of oral administration while maintaining comparable efficacy to intravenous fluoropyrimidines. What’s more, we retrospectively summarized an encouraging pCR rate of 38.1% of albumin-paclitaxel, cisplatin combined with capecitabine (APC regimen) as the neoadjuvant chemotherapy for 21 patients of locally advanced ESCC. The incidence of grade 3/4 neutropenia and leukopenia was 35.5% and 9.7%, respectively^[^17^]^. This data provided a strong rationale for evaluating the APC regimen in a prospective clinical trial. We launched a prospective phase 2 clinical trial in May 2020 to further verify the effectiveness and safety of the APC regimen as neoadjuvant chemotherapy for ESCC. Here we report the results with a median follow-up time to date exceeding 3 years.

## Materials and methods

Our trial was a single-center, prospective phase 2 clinical study in patients with locally advanced resectable esophageal squamous carcinoma to evaluate the efficacy and safety of the APC regimen as neoadjuvant chemotherapy. The research protocol (Supplemental Digital Content File 1, available at: http://links.lww.com/JS9/E114) was approved by the ethics committee of the Cancer Hospital Chinese Academy of Medical Sciences (20/058-2254). This study has been reported in accordance with the STROCSS criteria^[^18^]^.

### Eligibility

The primary inclusion criteria were: (1) histologically or cytologically confirmed ESCC; (2) clinical staging of T2-4aN±M0 (according to American Joint Committee on Cancer [AJCC]/Union for International Cancer Control/tumor, node, metastasis [TNM] staging system 8th edition); (3) patients who had no history of antitumor therapy; (4) 18–75 years old; (5) an Eastern Cooperative Oncology Group (ECOG) score of 0–1. The primary exclusion criteria were patients with supraclavicular lymph node metastasis; celiac lymph node metastasis except for pericardial; and left gastric lymph node metastasis. All patients provided written informed consent prior to enrollment in the trial.

### Treatment

All the patients were scheduled to be treated with an APC regimen before surgery. Specific drug dosages were albumin-bound paclitaxel 125 mg/m^2^, on day 1 and day 8; cisplatin 60 mg/m^2^ on day 1 or divided into 2 days; capecitabine 875 mg/m^2^, po bid, from day 1 to day 14; 21 days for 4 cycles. The efficacy was evaluated by enhanced CT of the neck, chest, and abdomen every two cycles. Patients underwent radical esophagectomy 4–6 weeks after cessation of the last dose of neoadjuvant treatment. After surgery, the patient continued to receive the original regimen of chemotherapy for two cycles, depending on the pathology and the patient’s tolerance to the treatment. The criteria for determining eligibility for surgery post-chemotherapy included clinical assessment of tumor response, performance status (ECOG 0–1), general health and organ function, and surgical feasibility as evaluated by a multidisciplinary team.

### Endpoint

The primary endpoint of the study was the pCR rate. The secondary endpoints were the major pathological response (MPR) rate, radical resection rate (R0 resection rate), disease-free survival (DFS), event-free survival (EFS), OS, safety, and postoperative complications. The Becker standard was used to evaluate pathological regression of the primary tumor after surgery. No residual tumor cells were defined as type 1a, less than 10% were defined as type 1b, 10%–50% were defined as type 2, and the remainder were defined as type 3. Pathological remission assessed at grades 1a and 1b was MPR (including pCR), while pCR was defined as the absence of residual tumor cells (including primary tumors and lymph nodes). All pathological assessments were confirmed by two pathologists independently. EFS was defined as the time between initiation of neoadjuvant chemotherapy and the following events: progression of disease and/or toxicity that preclude surgery, local or distant recurrence after surgery, or death due to any cause. OS was defined as the time between the start date of neoadjuvant chemotherapy and the death of the patient from any cause. DFS was defined as the time from surgery to the first date of tumor recurrence or metastasis, or death of the patient due to any cause.

The evaluation systems used in this study included: the 8th edition AJCC TNM staging^[^8^]^; the U.S. Department of Health and Human Services Common Terminology Rating Criteria for Adverse Events version 5.0^[^19^]^; and the Clavien–Dindo postoperative complications classification^[^20^]^

### Statistical analysis

With a pCR rate of 5% for CF in the JCOG9907 study^[^9^]^, 17% for DCF in the phase 2 study^[^11^]^, and 10% for DCS in the phase 2 study^[^21^]^, we adopted a pCR rate of 15% as the historical control. Taking into consideration the results of our previous retrospective study (pCR rate 38.1%), it was expected that the pCR could be increased to 28% with a two-sided test, *α* = 0.05 and 1 – *β* = 0.8. A cohort size of 69 cases was needed, and with an expected 10% shedding rate, a minimum cohort size of 76 cases was required. Chi-square tests were used for statistical analyses of categorical variables (e.g. pCR and non-pCR). Independent *t*-tests or Mann–Whitney *U* tests were applied for continuous variables (e.g. tumor stage, nodal status), depending on data distribution.

## Results

### Baseline characteristics

**Figure 1.**
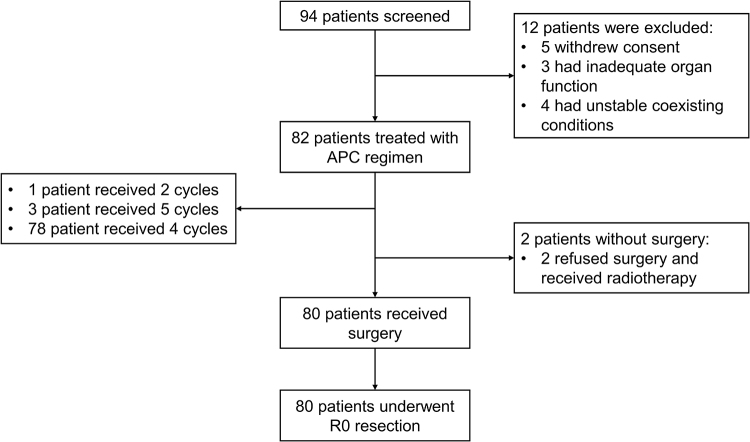
Consort flow diagram. APC, albumin-paclitaxel, cisplatin combined with capecitabine.

From May 19, 2020 to September 1, 2022, a total of 94 patients were screened (Fig. 1), among whom 82 patients with locally advanced ESCC were included in the present trial (Table 1). Of these, 75 were male and 7 females with a median age of 55 years (range: 42–75). The primary lesions were in the upper segment in 6 cases, middle segment in 30 cases, and lower segment in 46 cases. Forty-six patients had an ECOG score of 0 and 36 a scores of 1. Patients with T3 or T4a tumors accounted for 75.6% (62 cases) and 62 patients (75.6%) had positive lymph nodes.

**Table 1:** Patient demographics and disease characteristics (*N* = 82)

Characteristic	*N* (%)
Age, years	
Median (range)	55 (42–75)
Gender	
Male	75 (91.4)
Female	7 (8.6)
ECOG PS	
0	46 (56.1)
1	36 (43.9)
Clinical T category	
T2	20 (24.4)
T3	55 (67)
T4a	7 (8.6)
Clinical N category	
N0	20 (24.4)
N1	15 (18.3)
N2	47 (57.3)
cTNM staging	
II	23 (28)
III	52 (63.4)
Iva	7 (8.6)
Position	
Upper	6 (7.4)
Middle	30 (36.5)
Lower	46 (56.1)

### Treatment

All enrolled patients underwent preoperative chemotherapy, with a median of 4 (2–5) cycles of treatment. Cisplatin was administered over 2 days. Only 1 patient opted for surgery after 2 cycles of preoperative chemotherapy, while 3 patients received 5 cycles of preoperative chemotherapy. No patients experienced disease progression during preoperative chemotherapy.

Eighty patients underwent curative esophageal carcinoma resection. Two patients refused to undergo surgery and received radiotherapy after chemotherapy. Ten patients received adjuvant chemotherapy with the same regimen as preoperative chemotherapy, and no patient received adjuvant radiotherapy postoperatively.

### Surgical and postoperative pathology results

The R0 resection rate was 100% in 80 patients who underwent surgery. Twenty-three patients achieved pCR, of whom 17 were ypT0N0 and 6 ypTisN0, with a pCR rate of 28.7% (95% confidence interval [CI]: 18.8%–38.6%). Fifty patients achieved MPR, with an MPR rate of 62.5% (95% CI: 51.9%–73.1%). Forty-two (52.5%) patients had a postoperative pathological stage I (ypT0-2N0). Twenty-seven (33.8%) patients had postoperative N stage of N+, with a significant decrease from the preoperative percentage (75.6%). Only four patients had a postoperative stage N2. The T-stage of the primary lesion remained T3 or T4a in 21 patients (26.2%) after surgery, with a decrease from the preoperative percentage (75.6%) (Table 2). Among clinical and demographic factors, only the T category (tumor size) significantly correlates with pCR, while other factors like gender, age, and tumor location show no significant impact on pCR or MPR outcomes (Supplemental Digital Content Table 1, available at: http://links.lww.com/JS9/E115).

**Table 2:** Summary of the outcomes of surgery

Variables	*N* (%)
All patients received surgery (*n* = 80)	
pCR	23/80 (28.7)
MPR	50/80 (62.5))
R0 resection	80/80 (100)
Stage I (ypT1-2N0)	42 (52.5)
Stage II (ypT3N0)	11 (13.7)
Stage IIIA (ypT0-2N1)	15 (18.8)
Stage IIIB (ypT3N1/T0-3N2/T4a N0)	11 (13.7)
Stage IVA (ypT4aN2)	1 (1.3)
ypT0	22 (27.5)
ypTis	10 (12.5)
ypT1	18 (22.4)
ypT2	9 (11.3)
ypT3	20 (25)
ypT4a	1 (1.3)
ypN0	53 (66.2)
ypN1	23 (28.7)
ypN2	4 (5.1)

### Safety

During neoadjuvant chemotherapy, common adverse events were leukocytosis and neutropenia, as well as gastrointestinal symptoms such as nausea, vomiting, and loss of appetite, which were mostly grade 1–2 (Table 3). The most common grade 3–4 treatment-related adverse events were neutropenia (25.6%), leukocytosis (14.5%), nausea (7.5%), and peripheral neurotoxicity (5.5%). A total of six patients (7.3%) had their regimens adjusted due to adverse events, including five patients due to grade 4 neutropenia and one patient due to grade 2 peripheral neurotoxicity. Five patients developed complications within 1 month after surgery, including pneumonia (five patients, 6.3%), anastomotic fistula (one patient, 1.3%), and laryngeal recurrent nerve paralysis (one patient, 1.3%), all of which were grade 2. During preoperative chemotherapy and postoperative recovery, all adverse reactions were effectively managed, and there were no cases of treatment delay due to adverse reactions. There were no associated deaths during neoadjuvant chemotherapy or within 30 days after surgery.

**Table 3:** Summary of adverse events elicited by chemotherapy (*N* = 82)

Events	Any grade *N* (%)	Grade 3 *N* (%)	Grade 4 *N* (%)
Total	82 (100)	20 (24.4%)	3 (3.6%)
Neutropenia	65 (79.2%)	18 (21.9%)	3 (3.6%)
Leukopenia	66 (80.5%)	12 (14.5%)	0
Anemia	37 (45.1%)	0	0
Thrombocytopenia	19 (23.2%)	4 (4.8%)	0
Elevated ALT	18 (21.9%)	0	0
Elevated bilirubin	5 (5.5%)	0	0
Alopecia	62 (75.6%)	0	0
Nausea	60 (73.1%)	6 (7.5%)	0
Peripheral neurotoxicity	48 (58.5%)	5 (5.5%)	0
Loss of appetite	42 (51.2%)	0	0
Fatigue	22 (26.8%)	0	0
Vomiting	20 (24.3%)	0	0
Foot-hand syndrome	16 (19.5%)	0	0
Diarrhea	12 (14.6)	0	0

### Survival

**Figure 2.**
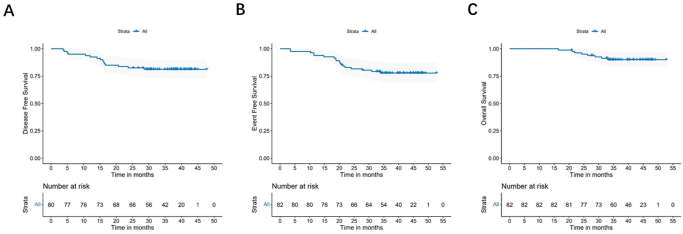
Kaplan–Meier estimates of disease-free survival, event-free survival, and overall survival. (A) Disease-free survival for patients after resection. (B) Event-free survival in the intention-to-treat population. (C) Overall survival in the intention-to-treat population.

Until May 31, 2024, the median follow-up time was 41.8 months, with local recurrence observed in 8 (8.5%) patients and distant metastasis was observed in 10 (12.2%) patients. Five patients had lung metastasis, four had liver metastasis, two had bone metastasis, two had retroperitoneal lymph node metastasis and one patient had brain metastasis. The 3-year DFS rate was 81.2%, the 3-year EFS rate was 77.8% and the 3-year survival rate was 90% (Fig. 2).

## Discussion

In this study, the combination of albumin paclitaxel, cisplatin, and capecitabine as neoadjuvant chemotherapy produced encouraging postoperative pathological remission and significant tumor down staging in patients with locally advanced ESCC. The four-cycle APC regimen achieved a pCR rate of 28.7% and an MPR rate of 62.5%, slightly lower than the findings of our previous retrospective study (with a pCR rate of 38.1% and an MPR rate of 71.4%). Moreover, 42 (52.5%) patients achieved postoperative tumor down staging of stage I (ypT0-2N0). The proportion of patients with staging still being N+ or T3/4a significantly decreased after surgery (N+ decreased from a preoperative 75.6% to a postoperative 33.8%; T3/4a decreased from a preoperative 75.6% to a postoperative 26.2%).

The pCR rate in this study was higher than those reported for CF and taxane-based regimens in prior trials. In the JCOG 1109 study, CF achieved a pCR rate of only 2%^[^6^]^, while paclitaxel combined with cisplatin in the ESCORT-NEO study achieved 4.7%^[^22^]^. Other regimens, including DCF, modified DCF, and DCS, reported pCR rates of 10%–19.8% in phase 2 and 3 trials. A two-cycle paclitaxel-CF regimen achieved a pCR rate of 24.1%, comparable to our findings, but with a 5-year OS rate of only 22%. In this study, the APC regimen also achieved encouraging survival, with a 3-year DFS rate of 81.2% and a 3-year OS rate of 90%. The JCOG 1109 study reported a 19.8% pCR rate and 3-year OS rate of 72.1% with DCF^[^6^]^, with updated 5-year OS and PFS rates of 65.1% and 55.7%, respectively, presented at the 2024 ASCO conference. These results suggest the APC regimen achieves not only a competitive pCR rate, but also promising survival outcomes. We also plan to conduct further follow-up, which will confirm the 5-year survival impact of the APC regimen.

Concurrent chemoradiotherapy with carboplatin/paclitaxel or cisplatin/vinorelbine both achieved impressive pCR rates of 49% and 43.2%, with 3-year OS rates of 58% and 69.1%, respectively in the CROSS and NEOCRTEC5010 studies. Similarly, in the JCOG1109 study, the concurrent chemoradiotherapy group achieved a pCR rate of 38.5% and a 3-year OS rate of 68.3%^[^6^]^. Although the pCR rate in our trial was not as good as the concurrent chemoradiotherapy mentioned above, the 3-year OS and DFS rates were encouraging. According to the results of neoadjuvant therapy studies for EC, although the pCR rate of neoadjuvant chemotherapy alone was lower than that of concurrent chemoradiotherapy, comparable OS and DFS still could be achieved. In addition to the JCOG1109 study, patients with ESCC in the CMISG1701 study^[^23^]^ were treated with four cycles of cisplatin (25 mg/m^2^, weekly) plus paclitaxel (50 mg/m^2^, weekly) combined with concurrent chemoradiotherapy followed by minimally invasive esophagectomy or were treated with two cycles of cisplatin (75 mg/m^2^, triweekly) plus paclitaxel (135 mg/m^2^, triweekly) followed by minimally invasive esophagectomy. Although the pCR rate of the chemoradiotherapy was significantly higher than that of chemotherapy alone (27.2% vs. 2.9%, *P* < 0.01), there was no significant difference in the 3-year survival rate (64.1% vs. 54.9%, *P* = 0.28) or PFS. The NEO-AEGIS phase 3 randomized controlled trial^[^24^]^ conducted in Europe also verified that the survival results of perioperative chemotherapy (MAGIC model) or neoadjuvant concurrent chemoradiotherapy (CROSS model) plus surgery in esophagus and esophagogastric junction adenocarcinoma were equivalent, the 3-year OS rates being 57% and 56%, respectively. Compared with neoadjuvant chemotherapy treatment alone, the reason why the higher pCR rate of concurrent chemoradiotherapy failed to provide a survival advantage may be partly related to the higher incidence of postoperative/long-term complications. Meta-analysis found that concurrent chemoradiation had a higher risk of postoperative death compared to neoadjuvant chemotherapy (relative risk [RR] = 1.58, 95% CR: 1.00–2.49)^[^25^]^.

Although in several single-arm phase 2 studies, the pCR rate of chemotherapy combined with ICIs could be as high as 51.1%, the pCR of camrelizumab combined with nab-paclitaxel and carboplatin was 28% in the latest ESCORT-NEO phase 3 clinical study, and the MPR was 59.1%. However, the OS results are not yet mature. Whether the survival benefit of the APC regimen is not inferior to that of concurrent chemoradiotherapy or chemotherapy plus an ICI needs further investigation. Interestingly, also in the ESCORT-NEO study, the pCR rates for paclitaxel or albumin-bound paclitaxel combined with cisplatin and camrelizumab were 28% and 15.4%, respectively, suggesting that albumin-bound paclitaxel may be a more effective partner for immunotherapy^[^22^]^.

In the present trial, the combination treatment of nab-paclitaxel, cisplatin, and capecitabine had a favorable safety profile, with neutropenia (25%), nausea (7.5%), and peripheral neurotoxicity (5.5%) being the main ≥ grade 3 adverse events. Of the enrolled patients, 98% completed the preplanned four cycles of neoadjuvant chemotherapy. The moderate dose intensity of albumin-bound paclitaxel (125 mg/m^2^, d1, 8) and cisplatin (60 mg/m^2^) may partly have contributed to the favorable safety. In the JCOG1109 study, DCF was administered at a higher dose (cisplatin 70 mg/m^2^, d1, docetaxel 70 mg/m^2^, d1 and 5FU 750 mg/m^2^ d1–5), which approached the original dose when first used in advanced gastric cancer studies (V325 study, 2006)^[^26^]^. The incidence of ≥ grade 3 neutropenia was 85.2% in the DCF group and the completion rate of the preplanned two cycles of chemotherapy was 85.6%. In the present trial, neoadjuvant treatment with APC followed by surgery also led to a lower incidence of postoperative complications. The most common complication was pneumonia (5/80, 6.3%) of which the incidence seemed lower than that of 10.9%–12.8% in chemoradiation studies^[^4,6^]^. While only 1 patient had an anastomotic fistula (1 case, 1.3%), of which the incidence can be 12.4% after chemoradiation and 10.3% after DCF (JCOG 1109 study)^[^6^]^. These results suggest that the APC regimen could serve as an effective alternative for neoadjuvant chemotherapy, particularly for patients who may not tolerate the higher toxicity of concurrent chemoradiotherapy.

At the present time, a variety of novel neoadjuvant treatment strategies including chemotherapy plus ICIs, chemoradiotherapy with ICIs, total neoadjuvant therapy (TNT), are in development and could be compatible with the current standard care in the near future for locally advanced ESCC. Each strategy may benefit a particular population of patients. Therefore, active exploration of predictive markers of efficacy and safety will be very critical. Taking into consideration molecular markers, the disease and specific patient characteristics will help in the development of an optimal individualized neoadjuvant treatment for each patient.

There were some limitations to the present trial. First, it was a single arm study with no control arm of concurrent chemoradiation, and the sample size was still not large enough. Second, the follow-up time was not long enough to obtain a 5-year survival rate. Finally, we did not explore therapeutic biomarkers.

## Conclusion

The neoadjuvant treatment with albumin-bound paclitaxel, cisplatin, and capecitabine is highly active with excellent pCR rates and promising survival in locally advanced ESCC and was well tolerated by patients. This combination could serve as one optional neoadjuvant chemotherapy regimen and warrants further investigation.

## Data Availability

The dataset supporting the conclusions of this article can be accessed upon reasonable request to the corresponding author. For any restrictions related to ethical or legal concerns, access may be granted only after appropriate approvals.

## Research registration unique identifying number (UIN)

The study is registered on ClinicalTrials.gov with registration number NCT04390958.
